# P-204. Contemporary Economic and Healthcare Burden Associated with *Clostridiodes difficle* Infections (CDI) in the United States

**DOI:** 10.1093/ofid/ofae631.408

**Published:** 2025-01-29

**Authors:** Dipendra Thapaliya, Vidya Krishnan, Hannah Hill

**Affiliations:** Case western Reserve University/MetroHealth Medical Center, Cleveland, Ohio; MetroHealth Medical Center, Cleveland, Ohio; MetroHealth Medical Center, Cleveland, Ohio

## Abstract

**Background:**

Centers for Disease Control and Prevention (CDC) estimated that in 2017, there were 223,900 cases of CDI in hospitalized patients and 12,800 deaths in the United States. CDI is the most common health care-associated infection, and a leading cause of diarrhea in the hospitalized patients (Magill et. al., 2015). Incidence of Community-associated CDI is rising in recent years (Chitnis et. al., 2013; Gerding & Lessa 2015).

**Figure:**
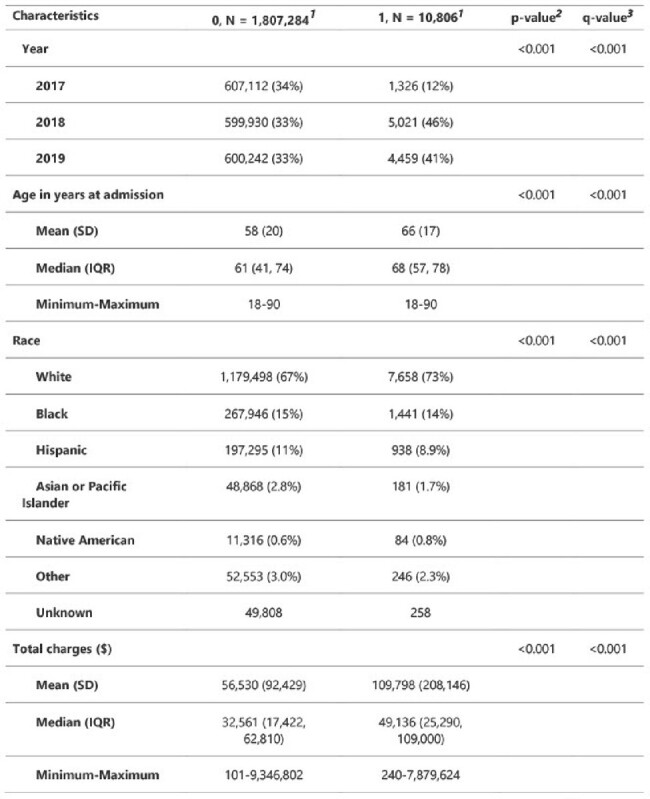

**Methods:**

The National Inpatient Samples (NIS) datasets include de-identified patient-specific and hospital-specific information for hospitalized patients across the U.S. We utilized these datasets to study the economic and healthcare burden associated with CDI. Data were downloaded and SAS software was used for analyses. Continuous variables were summarized by means and standard deviations and compared using Student’s t-tests. Categorical variables were summarized by percentages and compared using Chi-squared or Fisher’s tests, as appropriate. Bivariate and multivariable logistic regression models were created to determine factors that are associated with the primary outcome.

**Table 1:**
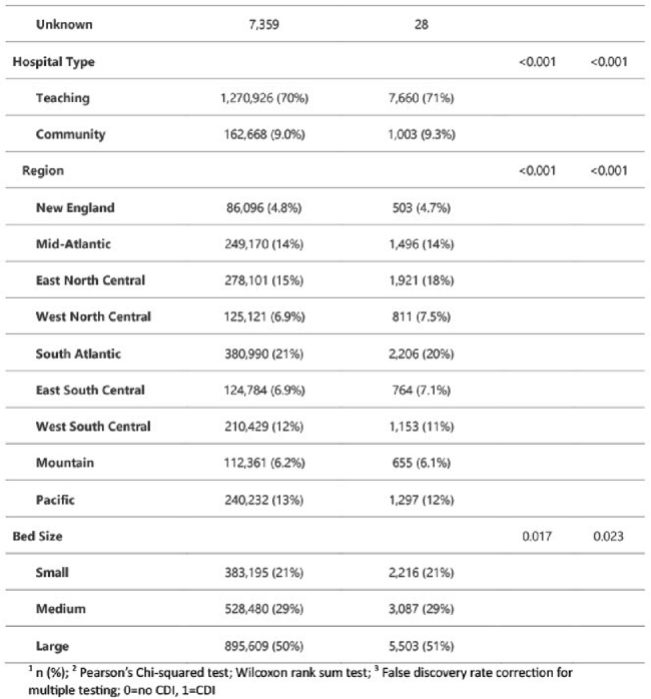
Patient Characteristics

**Results:**

A total of 10,806 cases of CDI were identified among in-patient samples of 1818,090 patients during 2017-2019 in 9 geographic regions across the United States. This constitutes 10% sample of the NIS dataset. Compared to 2017, significant increase in CDI was observed in 2018 and 2019 (12% vs 46 and 41% respectively). Of the patient population, mean age at admission was 66 years; white had the highest cases of CDI compared to any other race (p < 0.001). Majority of the CDI cases were observed in teaching hospitals compared to community hospitals (71% vs 9.3%; p=< 0.001). South Atlantic region had the highest CDI burden (20%) followed by East North Central region (18%). Large hospitals based on bed size observed significantly higher cases of CDI (51%, p = 0.017). Hospitalization charges was significantly higher among CDI patients (mean $ 109,798 vs $56,530; p = < 0.001).

**Conclusion:**

These results highlight the significant disease morbidity and mortality as well as economic burden caused by CDI and warrants effective mitigation strategies to address the risk factors to minimize CDI.

**Disclosures:**

**All Authors**: No reported disclosures

